# Annotations

**Published:** 1906-08-04

**Authors:** 


### ANNOTATIONS.

### The B.M.A. and the General Medical Council.

In a recent issue we expressed a strong opinion against the proposal on the part of certain leading spirits of the British Medical Association to demand a restrictive pledge from candidates to be nominated by the Association for election to the General Medical Council. Such a procedure, we pointed out, was worthy of a political caucus rather than of a liberal profession, and was calculated to prejudice the cause of direct representation in the Council by reducing those elected to the position of delegates in the service of an external and irresponsible organisation. That our protest was well founded was made very evident by what occurred last week at the annual representative meeting of the Association. First, the discussion showed that a very strong feeling had been excited in the profession by the attempt to enforce on candidates a restriction of their liberty of action. Again, specific declarations were made to the effect that certain members of the profession who enjoy a large measure of respect and confidence had declined to accept the pledge as demanded by the officials, and, as a consequence, their names did not appear in the voting-papers. Further, it was shown that any member elected to the Medical Council under the conditions suggested by the Association would be liable to be excluded when certain kinds of business came up for consideration. Under the weight of these arguments the honeyed definition of the pledge as "merely an expression of loyalty" was promptly strained to the breaking point, and with this collapse the entire scheme fell to the ground. Thus, not only the pledge, but also the proposal that the Association should run official candidates, disappears, and we think the disappearance a fortunate event. Certainly it is a plain hint to those who seem to think that medical practitioners can be schooled in the narrow principles of compulsory trades unionism.

### Perils of the Foreshore.

The holiday season, as frequent warnings remind us, has its peculiar perils and contributes its special list of victims to disease and death. Even the invigorating pleasures of the foreshore are mingled with disastrous possibilities for the careless and unwary. Fortunately, just as danger lies in ignorance, safety is to be found in knowledge. An apt illustration of this position may be found in the last report of Dr. W. S. Cook, medical officer of health for Greenock. In this he expresses the opinion that a distinct danger to the public arises from the use as food of shell-fish and edible sea-weed gathered on the foreshore. He quotes several outbreaks of enteric fever which have been traced to shell-fish gathered in the vicinity of sewage outlets, and describes the actual discovery of the *Bacillus typhosus* in specimens collected from popular resorts. Among visitors to these resorts it is a popular practice to wander on the foreshore and to consume shell-fish picked up by themselves. There is a pleasing belief that, collected in this manner, the shell-fish must needs be "fresh," and Dr. Cook quotes an ingenuous youth who informed him that "the best place for dulse is near the pipe"—meaning the sewer from the main drainage system of the town. It is undoubtedly the duty of the local authorities to post notices warning visitors against the indiscriminate consumption of shell-fish collected on the beach. Need for official supervision is further emphasised by a recent outbreak of enteric fever at West Ham, in which of 52 patients 24 were found to have eaten cockles a short time before the attack. The incriminated cockles have been traced to Leigh-on-Sea, and the City Corporation has very properly called upon dealers to discontinue the sale of cockles from this source. But no policy will be entirely successful that does not strike at the source of contamination. The belief that "boiling"—in the domestic interpretation of this term—will neutralise the danger of sewage pollution is not well founded.

### A Public Medical Service.

A recent article in the *Midland Medical Journal* brings to notice once more the present unsatisfactory status of public medical charities. There is, without question, a growing feeling in the mind of the working-classes that the hospital service to which they have grown accustomed is not a favour, but a right. This gradual assumption of property in what took origin as an act of grace is as common in private relations as in this public one, but it is always an unpleasing phenomenon, and never more so than in the present instance. It gives to the recipients an air of ingratitude which is galling in the extreme to the executive of charities, who are often performing their onerous duties to a large extent as an act of personal charity and sacrifice. None the less this attitude is not surprising, for the enormous growth of medical charities has led their administrators to make their appeals for support as comprehensive as possible. Thus the working-classes are invited to subscribe their mites to the support of hospitals, and they respond to a varying extent. But having responded, they look upon their subscription not as a free gift, or act of sacrifice, but as a kind of insurance against ill-health in the future; they look upon themselves as the supporters of the charity whose support has given them a prescriptive right to the enjoyment of its benefits, and this whether their financial position justifies the attitude or not. In consequence the private practitioner suffers, and the honorary medical staffs of hospitals are faced with a mass of work quite disproportionate to their numbers. It is becoming recognised that drastic reforms in the mode of admission and in cutting down the numbers are imperatively called for, reforms which have only been postponed so long by the extraordinary philanthropy of the rich, and the forbearance and submission to "sweating" of the honorary medical officers of hospitals. We do not claim for the latter any extraordinary altruism. They seek their appointments largely because they desire the experience which flows from hospital work, but the value of such appointments becomes almost nil when the numbers to be dealt with become extreme, and the burden thus shorn of interest is almost intolerable.

